# Transferring knowledge of bacterial protein interaction networks to predict pathogen targeted human genes and immune signaling pathways: a case study on *M. tuberculosis*

**DOI:** 10.1186/s12864-018-4873-9

**Published:** 2018-06-28

**Authors:** Suyu Mei, Erik K. Flemington, Kun Zhang

**Affiliations:** 1 Software College, Shenyang Normal University, Shenyang, 110034 China; 2 Department of Pathology, Tulane Cancer Center, Tulane University, New Orleans, LA 70112 USA; 3 Department of Computer Science, Bioinformatics facility of Xavier NIH RCMI Cancer Research Center, Xavier University of Louisiana, New Orleans, LA 70125 USA

**Keywords:** Pathogen-host protein interaction networks, Pathogen-host coevolution, Signaling pathways, Transfer learning, l_2_-regularized logistic regression

## Abstract

**Background:**

Bacterial invasive infection and host immune response is fundamental to the understanding of pathogen pathogenesis and the discovery of effective therapeutic drugs. However, there are very few experimental studies on the signaling cross-talks between bacteria and human host to date.

**Methods:**

In this work, taking *M. tuberculosis* H37Rv (MTB) that is co-evolving with its human host as an example, we propose a general computational framework that exploits the known bacterial pathogen protein interaction networks in STRING database to predict pathogen-host protein interactions and their signaling cross-talks. In this framework, significant interlogs are derived from the known pathogen protein interaction networks to train a predictive l_2_-regularized logistic regression model.

**Results:**

The computational results show that the proposed method achieves excellent performance of cross validation as well as low predicted positive rates on the less significant interlogs and non-interlogs, indicating a low risk of false discovery. We further conduct gene ontology (GO) and pathway enrichment analyses of the predicted pathogen-host protein interaction networks, which potentially provides insights into the machinery that *M. tuberculosis* H37Rv targets human genes and signaling pathways. In addition, we analyse the pathogen-host protein interactions related to drug resistance, inhibition of which potentially provides an alternative solution to *M. tuberculosis* H37Rv drug resistance.

**Conclusions:**

The proposed machine learning framework has been verified effective for predicting bacteria-host protein interactions via known bacterial protein interaction networks. For a vast majority of bacterial pathogens that lacks experimental studies of bacteria-host protein interactions, this framework is supposed to achieve a general-purpose applicability. The predicted protein interaction networks between *M. tuberculosis* H37Rv and *Homo sapiens*, provided in the Additional files, promise to gain applications in the two fields: (1) providing an alternative solution to drug resistance; (2) revealing the patterns that *M. tuberculosis* H37Rv genes target human immune signaling pathways.

**Electronic supplementary material:**

The online version of this article (10.1186/s12864-018-4873-9) contains supplementary material, which is available to authorized users.

## Background

Bacterial invasive infection and host immune response is fundamental to the understanding of pathogen pathogenesis and the discovery of effective therapeutic drugs. As an example, *Mycobacterium tuberculosis* is the causative agent of tuberculosis, an infectious disease that causes millions of deaths each year [1]. In recent years, *M. tuberculosis* H37Rv has attracted much attention partly due to its co-infection with HIV [2] and drug resistance [3–6]. From the point of view of interactome, bacterial-host protein interaction networks can be viewed as the interface/cross-talks between pathogen protein-protein interaction (PPI) networks and host PPI protein-protein networks. Bacteria-host signaling cross-talks potentially help us understand the underlying mechanism of *M. tuberculosis* infection and human defence.

To date, most of the experimental work focuses on detecting protein-protein interactions within bacterial cells. The database STRING [7] (https://string-db.org/) has curated massive PPI networks of 1678 bacterial pathogens such as *M. tuberculosis*, *B. anthracis*, *F. tularensis*, *Y. pestis*, etc. However, there are very few experimental studies on protein interactions between bacteria and their host. From a computational view of point, *M. tuberculosis* H37Rv has been extensively studied in recent years in terms of drug resistance analysis [4–6, 8] and PPI networks reconstruction [9, 10]. In [9, 10], interlogs are derived as *M. tuberculosis* H37Rv PPIs from the known PPIs of other source species. In [9], the known *M. tuberculosis* H37Rv PPIs are laid aside unused and instead the *E. coli* PPIs are used as training data to predict *M. tuberculosis* H37Rv PPIs. In [10], the interlogs derived from *lostridium difficile* are used to expand the known *M. tuberculosis* H37Rv PPI networks*,* and the expanded PPI networks are further used as training data to train a random forest model for the discovery of novel *M. tuberculosis* H37Rv PPIs.

Bacterial pathogen PPI networks are useful to study the signaling mechanism and drug resistance machinery within bacteria cell. However, we need further reconstruct bacteria-host PPI networks to understand the cross-talk mechanism of bacterial infection and host immunity. In recent years, pathogen-host PPI networks reconstruction and pathogen-host signaling cross-talk modeling have attracted much attention from computational biologists [11–18], most of which focus on virus-host protein interactions. Comparatively, t the experimental studies on bacteria-host protein interactions are much less than that on virus-host protein interactions, partly because of the complex bacterial cell wall, which forms a strong permeability barrier to the mutual access of the bacterial genome and the host genome [19]. The two genomes could come across to physically interact only if bacterial proteins are located at the surface or membrane of bacterial cell, or bacterial proteins could transport or secret into the host cell. To our knowledge, experimental studies on bacteria-host protein interactions have been conducted for a very limited number of species such as *Salmonella* [20], *Bacillus anthracis*, *Francisella tularensis*, and *Yersinia pestis* [21]. For these bacterial pathogens, the known pathogen-host PPIs can be used as training data of machine learning modeling or be treated as templates to infer interlogs [22]. In [22], the known 62 *Salmonella*-human PPIs are used to derive interlogs as novel *Salmonella*-human PPIs.

Nevertheless, no experimental studies on pathogen-host protein interactions have been conducted for the overwhelming majority of bacteria, e.g. *M. tuberculosis* H37Rv. To study the signaling cross-talks between bacteria and host, two solutions to inferring bacteria-host protein interactions have been proposed, one solution is ortholog knowledge transfer that transfers ortholog knowledge between two different hosts, e.g. knowledge transfer between human and plant to infer *Salmonella*-plant PPIs from the known *Salmonella*-human PPIs [23]; and the other solution is interlog knowledge transfer that infers interlogs from known PPIs of different bacteria and different hosts [24, 25]. These two solutions are effective for cross-species knowledge transfer, especially when no experimental data are available to the species to be studied. Nevertheless, these two methods both resort to third-party species that may not physically co-exist with parasitic relationships, e.g. *Homo sapiens* versus plant [23]. Ortholog or interlog knowledge transfer across widely-variant species are prone to yield a certain level of noise and false interactions due to a large evolutionary divergence.

Actually, the parasitic or co-evolution relationships between bacteria and host indicate that bacterial protein-protein interaction alone is sufficient for us to infer bacteria-host protein interaction without resorting to third-party distant species. Knowledge transfer between two co-evolving species is more credible than that between two evolutionarily distant species. The bacterial pathogen protein interaction networks of 1678 bacteria in STRING [7] provide rich information for us to study bacteria-host protein interactions, because many evidences have demonstrated that bacterial genome is co-evolving with its host genome [19, 26, 27]. In [27], it has been concluded that *Mycobacterium tuberculosis* complex (MTBC) has been anatomically co-evolving with modern humans for tens of thousands of years on the basis of the evidences of its origin in Africa, the congruence in phylogeography and the dating of major branching events. Moreover, the drug resistance of *Mycobacterium tuberculosis* (MTB) strains is also evolving with the host genome. In [19], it has been claimed that human genetic factors may play important roles in MTB drug resistance and different MTB lineages (e.g. lineage 1: Indo-Oceanic; lineage 2: East Asian; lineage 3: Central Asian; lineage 4: Euro American; lineage 7: Ethiopia) acquire different levels of drug resistance. Molecular interactions are an effective way to unravel bacteria-host co-evolution relationship and the progression of bacterial drug resistance, which is at present hampered by the limited knowledge of bacteria-host interactions. For instance, the molecular mechanism involved in sensing of extracellular signals for inducing its metabolic adaptation still remains unclear [4].

Furthermore, the interaction between bacteria and host also somewhat contributes to bacterial drug resistance. As suggested in [19], the interaction of MTB with its macrophage microenvironment may play an important role in the risk of progression to drug-resistant TB. Meanwhile from the host side, polymorphisms within genes involved in macrophage activity (*SLC11A1*, *VDR* and *HLA* genes) have been reported to be associated with susceptibility to MTB drug resistance. As reviewed in [28], the amino acid residues from the PPI interfaces are more conserved than those from other parts of the protein surface, and PPI inhibitors can perhaps be more resistant to spontaneous mutations at their binding site versus inhibitors of the active site, thus bacteria-host PPI inhibitors may be of particular interest as antimicrobial drugs that induce less risk of drug resistance. Therefore, the exploration of protein interaction networks between co-evolving bacterial pathogens and host could potentially achieve two goals: (1) deriving more reliable interlogs to study pathogen-host signaling cross-talks; (2) choosing pathogen-host PPI inhibitors to provide an alternative solution to bacterial drug resistance.

In this work, taking *M. tuberculosis* H37Rv as an example, we propose a general computational framework that transfers the knowledge of bacterial pathogen protein interaction networks to predict pathogen targeted human genes and immune signaling pathways. Due to lack of experimental studies, we take advantage of the co-evolution relationship between *M. tuberculosis* H37Rv and *Homo sapiens* [19, 26, 27] to derive interlogs as the training data from *M. tuberculosis* H37Rv protein interactions alone. We confine the search of *M. tuberculosis* H37Rv ortholog genes within its human host without resorting to a third-party species. The interlogs derived in this way are presumably more reliable than those derived from remote species. Given two interacting *M. tuberculosis* H37Rv genes (*m*_*1*,_ *m*_*2*_) and their corresponding *Homo sapiens* ortholog genes (*h*_*1*_, *h*_*2*_), we deem (*m*_*1*_, *h*_*2*_) and (*m*_*2*_, *h*_*1*_) as two interlogs, since the human ortholog gene products are functionally or structurally similar to *M. tuberculosis* H37Rv gene products. To ensure the quality of data, only the significant interlogs are used as training data, and the less significant interlogs need to be further validated by the trained model. Ortholog and interlog knowledge transfer are prone to introduce a certain level of noise as well as increase the computational complexity. To solve this problem, we adopt theoretically well-established l_2_-regularized logistic regression as the base machine learning model. Finally, we further conduct gene ontology (GO) and pathway enrichment analyses on the predicted interactions to provide insights into the machinery of *M. tuberculosis* H37Rv infection and host response. As a major concern in recent years, bacterial drug resistance is also discussed in terms of bacteria-host PPI inhibition to provide a potential alternative solution to *M. tuberculosis* H37Rv drug resistance.

## Methods

### Overview flowchart of the proposed framework

**Fig. 1:**
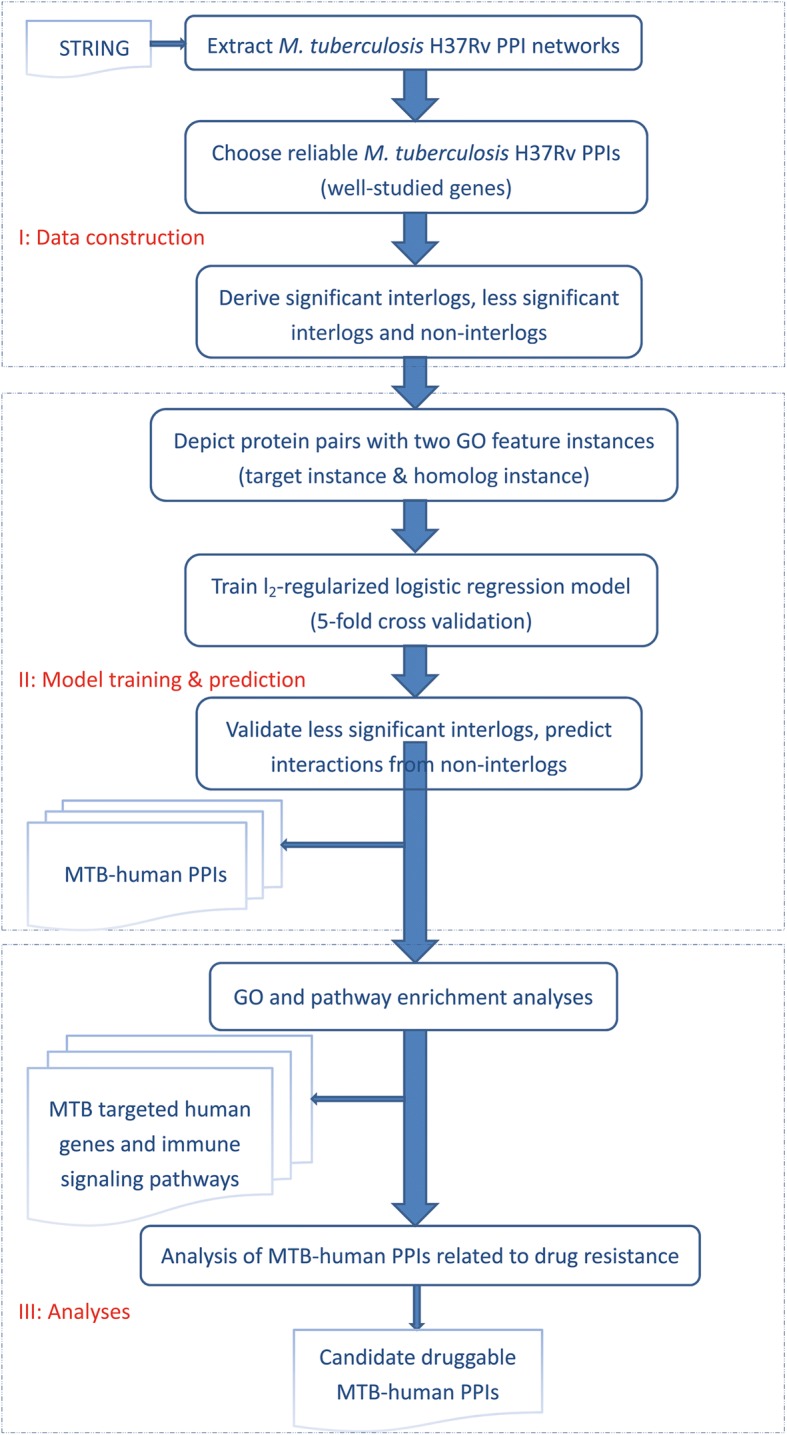
Overview flowchart of the proposed framework

As shown in Fig. 1, this work is divided into three phases: (I) data construction; (II) model training and prediction; (III) analyses. The critical phase is to derive interlogs as training data from the known *M. tuberculosis* H37Rv PPI networks in STRING [9]. This phase takes advantage of the co-evolution relationship between *M. tuberculosis* H37Rv and human host to construct training data that are experimentally not available. The second phase is to construct feature representation, train an l_2_-regularized logistic regression model, and then predict interactions from less significant interlogs and non-interlogs. The final phase is to comprehensively analyse the reconstructed MTB-human PPI networks for further understanding of the machinery of bacterial infection and host response. Especially, we analyse the MTB-human PPIs related to *M. tuberculosis* H37Rv drug resistance. Proper selection or design of PPI inhibitors could potentially provide an alternative solution to bacterial drug resistance.

### Data construction via interlog knowledge transfer from *M. tuberculosis* H37Rv PPI networks

The *M. tuberculosis* H37Rv protein interaction networks in STRING [9] contain 309,715 interactions. Unfortunately, the PPI networks have been estimated to be of low quality [24, 25, 29]. To choose quality *M. tuberculosis* H37Rv protein interactions, we take the following three measures. Firstly, we only choose the PPIs with experimental evidences. Secondly, we remove the proteins that have no gene names. Lastly, we only choose the well-studied genes that have been annotated with at least one specific gene ontology (GO) term of molecular function or biological process, except the generic root GO terms (GO:0005575, GO:0008150, GO:0003674) in the GO directed acyclic graph (DAG). As results, we obtain 5224 well-studied *M. tuberculosis* H37Rv genes that correspond to 7835 gene products/proteins and 28,347 *M. tuberculosis* H37Rv protein-protein interactions. In the same way, we obtain 20,081 well-studied human genes that correspond to 60,126 gene products/proteins.

Now we exploit the knowledge of *M. tuberculosis* H37Rv PPI networks via orthologous relationships to construct the training data for MTB-human PPI predictions*.* To formally formulate the method of data construction, we denote the obtained *M. tuberculosis* H37Rv PPI networks as *G,* which contains 28,347 interactions and 1469 well-studied *M. tuberculosis* H37Rv proteins. After removing the *M. tuberculosis* H37Rv proteins that have no human orthologs, we obtain 1359 well-studied *M. tuberculosis* H37Rv proteins in total. We search the human ortholog genes in SwissProt [30] simply using PSI-BLAST [31] with default E-value (E-value = 10). Orthologs are defined as homologous genes diverging after a speciation event [32]. Actually, there are some advanced methods to search or predict orthologs, e.g. Reciprocal Best Hits (RBH) [32, 33], which relies on BLAST [34] to identify pairwise orthologs between two species [33], that’s, two genes residing in two different genomes are deemed orthologs if their protein products find each other as the best hit in the opposite genome [32]. For each ortholog pair (A, A’), RBH algorithm needs to run BLAST twice in two directions, one direction is against A query genome, and the opposite direction is against A’ query genome. RBH algorithm yields quality orthologs at the cost of high computational intensity. In this work, we adopt simple homolog-search method instead of RBH algorithm for the two reasons: (1) we need more orthologs including the distant orthologs to derive interlogs as training data, because no experimental data are available to computational modeling; (2) the RBH algorithm would computationally worsen the efficiency of the sophisticated framework as illustrated in Fig. 1. If we choose lower BLAST E-value cutoff, e.g. 1e-50 versus 1e-6 [32], we still could obtain quality orthologs. The noise from distant orthologs or non-orthologs could be counteracted using the regularization technique that is discussed in the next subsection.

From *G*, we derive the interlogs as follows. Given two interacting *M. tuberculosis* H37Rv genes (*m*_*i*_, *m*_*j*_), we use *H*_*i*_, *H*_*j*_ to denote the sets of ortholog genes *m*_*i*_ and *m*_*j*,_ respectively. In particular, if an ortholog gene yields more than one ortholog protein, only one ortholog protein is randomly chosen as the interacting partner. Given the cutoff of ortholog significance *δ*, e.g. E-value of PSI-BLAST, we further split *H*_*i*_, *H*_*j*_ into two subsets $\left\{{H}_i^{\le \delta },{H}_i^{>\delta}\right\},\left\{{H}_j^{\le \delta },{H}_j^{>\delta}\right\}$, respectively. Here we define ${H}_i^{\le \delta },{H}_j^{\le \delta }$ as the sets of significant ortholog genes and ${H}_i^{>\delta },{H}_j^{>\delta }$ as the set of less significant ortholog genes. For any two interacting *M. tuberculosis* H37Rv genes (*m*_*i*_, *m*_*j*_), we create the positive training instances from the set of significant ortholog genes ${H}_i^{\le \delta },{H}_j^{\le \delta }$ as follows.

1 $$ Pos\left({m}_i,{m}_j\right)=\left\{\left({m}_i,\mathrm{g}\right)|\mathrm{g}\in {H}_j^{\le \delta}\right\}\cup \left\{\left({m}_j,\mathrm{g}\right)|\mathrm{g}\in {H}_i^{\le \delta}\right\} $$

where (*m*_*i*_, g) or (*m*_*j*_, g) denotes significant interlog. Formula (1) is based on the assumption that the *M. tuberculosis* H37Rv gene *m*_*i*_(*m*_*j*_) functionally or structurally co-evolves with its human host ortholog genes $\left\{\mathrm{g}|\mathrm{g}\in {H}_i^{\le \delta}\right\}$ ($\left\{\mathrm{g}|\mathrm{g}\in {H}_j^{\le \delta}\right\}$). The interaction of *m*_*i*_ with *m*_*j*_ to a great extent indicates the interaction of *m*_*i*_ with *m*_*j*_ ‘s ortholog gene $\left\{\mathrm{g}|\mathrm{g}\in {H}_j^{\le \delta}\right\}$, and the interaction of *m*_*j*_ with *m*_*i*_ ‘s ortholog genes $\left\{\mathrm{g}|\mathrm{g}\in {H}_i^{\le \delta}\right\}$ vice versa. All the positive training instances are then merged to generate the whole positive training set.

2 $$ {U}_{pos}=\underset{\left({m}_i,{m}_j\right)\in G}{\cup } Pos\left({m}_i,{m}_j\right) $$

From the evolutionary point of view, the less significant interlogs are not so reliable as the significant interlogs, so that they need to be further validated by the predictive model, which is trained on the significant interlogs. The set of less significant interlogs is defined as follows.

3 $$ {Val}_{pos}=\underset{\left({m}_i,{m}_j\right)\in G}{\cup}\left\{\left({m}_i,\mathrm{g}\right)|\mathrm{g}\in {H}_j^{>\delta}\right\}\cup \left\{\left({m}_j,\mathrm{g}\right)|\mathrm{g}\in {H}_i^{>\delta}\right\} $$

where (*m*_*i*_, g) or (*m*_*j*_, g) denotes insignificant interlog. For each *M. tuberculosis* H37Rv gene *m*_*i*_, let *P*_*i*_ = {*g*| {*m*_*i*_, *g*} ∈ *U*_*pos*_} denote the set of its human partner genes, *M*_*i*_ = {*g*| {*m*_*i*_, *g*} ∈ *G*} denote the set of its *M. tuberculosis* H37Rv partner genes, and $All\_{orth}_i=\underset{m_j\in {M}_i}{\cup }{H}_j$ denote the set of human ortholog genes of all the genes in *M*_*i*_. Then we randomly sample the human genes that potentially do not interact with gene*m*_*i*_ from the set non-ortholog genes *N*_*i*_ = {*g*| *g* ∉ *All*_*orth*_*i*_ ^ *g* ∈ *Homo*_*well*_} to construct the negative training data, where *Homo*_*well*_ denotes the well-studied human genes. To obtain well-balanced training data, we impose the constraint ∣*N*_*i*_ ∣  =  ∣ *P*_*i*_∣ on the sampling of negative training data. Then the whole negative training set is defined as follows.

4 $$ {U}_{neg}=\underset{m_i\in M}{\cup}\left\{\left({m}_i,g\right)|g\in {N}_i\right\} $$

where *M* denotes the set of *M. tuberculosis* H37Rv genes in the *M. tuberculosis* H37Rv PPI networks *G*.

Model evaluation is a hard problem in the case of lack of experimental data. Nevertheless, random sampling of a tiny fraction of data in the huge space of non-interlogs is convincingly to capture non-interactions with a large probability. Hence we obtain the negative data to estimate the model as follows. Let ${N}_i^{\hbox{'}}=\left\{g|g\notin All\_{orth}_i\hat{\mkern6mu} g\notin {N}_i\hat{\mkern6mu} g\in {Homo}_{well}\right\}$ denote the set of human genes, from which the negative data to be validated are defined as follows.

5 $$ {Val}_{neg}=\underset{m_i\in M}{\cup}\left\{\left({m}_i,g\right)|g\in {N}_i^{\hbox{'}}\right\},s.t.\mid {Val}_{pos}\mid =\mid {Val}_{neg}\mid $$

For each *M. tuberculosis* H37Rv gene *m*_*i*_, we sample the human genes from the set ${N}_i^{\hbox{'}\hbox{'}}=\left\{g|g\notin All\_{orth}_i\hat{\mkern6mu} g\notin {N}_i\hat{\mkern6mu} g\notin {N}_i^{\hbox{'}}\hat{\mkern6mu} g\in {Homo}_{well}\right\}$ to obtain the prediction set as follows.

6 $$ Pred=\underset{m_i\in M}{\cup}\left\{\left({m}_i,g\right)|g\in {N}_i^{\hbox{'}\hbox{'}}\right\} $$

The prediction set is further reduced for the sake of computational complexity by imposing a constraint on the space as follows.

7 $$ Pred=\underset{m_i\in M}{\cup}\left\{\left({m}_i,g\right)|g\in {N}_i^{\hbox{'}\hbox{'}}\right\},s.t.\mid {N}_i^{\hbox{'}\hbox{'}}\mid \le 300 $$

Formula (7) means that no more than 300 human proteins are randomly sampled for each *M. tuberculosis* H37Rv protein. The data and analyses are referred to the section *Results*.

### Multi-instance GO feature construction via homolog knowledge transfer

State-of-art feature construction is a critical step of machine learning modeling in solving specific problems. Gene ontology (GO) has been widely used as features to predict protein-protein interactions [14–18, 35–40]. In [35], GO has been claimed to be the most discriminative feature for PPI prediction [35]. Gene ontology is a hierarchically organized and controlled vocabulary that characterizes gene products [41], and the annotations of genes or gene products are provided in terms of GO terms in GOA [42]. Despite its powerful predictive capability, GO feature representation could encounter a serious problem for those less-studied or novel genes, because the sparsity of GO terms potentially yields null feature vectors. In this work, homolog knowledge transfer is conducted via independent homolog instances to solve this problem, that is, each gene/protein is depicted with two instances, namely target instance and homolog instance. The target instance depicts the GO knowledge of the gene/protein itself, and the homolog instance depicts the GO knowledge of the homologs. The homologs are extracted from SwissProt [30] using PSI-BLAST [31] (E-value = 10) against all species. We treat all types of evidence codes equally including ISS (Inferred from Sequence or structural Similarity), IEA (Inferred from Electronic Annotation), etc. The reason that we choose the default E-value is that we need to capture distant homologs. Similarly, the reason that we do not filter out those indirectly-derived or uncurated annotations is to overcome the sparsity and enlarge the coverage of GO terms. Undoubtedly, a certain level of noise would be introduced into the computational framework, which will be discussed in the next subsection. The GO terms are extracted from GOA [42]. For each protein *i* in the training set *U*, we obtain two sets of GO terms, one set denoted as ${S}_H^i$ contains the GO terms of the homologs, and the other set denoted as ${S}_T^i$ contains the GO terms of the protein itself. Then the whole set of GO terms of the training set is defined as follows:

8 $$ S=\underset{i\in U}{\cup}\left({S}_T^i\cup {S}_H^i\right) $$

For each protein pair (*i*_1_, *i*_2_), the target instance and the homolog instance are formally defined as follows:

9 $$ {V}_T^{\left({i}_1,{i}_2\right)}\left[g\right]=\left\{\begin{array}{c}0,g\notin {S}_T^{i_1}\wedge g\notin {S}_T^{i_2}\\ {}2,g\in {S}_T^{i_1}\wedge g\in {S}_T^{i_2};\\ {}1, otherwise\end{array}\right.{V}_H^{\left({i}_1,{i}_2\right)}\left[g\right]=\left\{\begin{array}{l}0,g\notin {S}_H^{i_1}\wedge g\notin {S}_H^{i_2}\\ {}2,g\in {S}_H^{i_1}\wedge g\in {S}_H^{i_2}\\ {}1, otherwise\end{array}\right. $$

For each GO term *g* ∈ *S*, ${V}_T^{\left({i}_1,{i}_2\right)}\left[g\right]$ denotes the component *g* of the target instance ${V}_T^{\left({i}_1,{i}_2\right)}$ and ${V}_H^{\left({i}_1,{i}_2\right)}\left[g\right]$ denotes the component *g* of the homolog instance ${V}_H^{\left({i}_1,{i}_2\right)}$. Those GO terms *g* ∉ *S* are discarded. Formula (9) means that if the protein pair (*i*_1_, *i*_2_) shares the same GO term *g*, then the corresponding component in the feature vector ${V}_T^{\left({i}_1,{i}_2\right)}$ or ${V}_H^{\left({i}_1,{i}_2\right)}$ is set 2; if neither protein in the protein pair possesses the *GO* term *g,* then the value is set 0; otherwise the value is set 1. The GO terms of the protein pair (*i*_1_, *i*_2_) that do not belong to the whole set of GO terms of the training set, formally defined as $\left\{g|g\in {S}_T^i\vee g\in {S}_H^i\wedge g\notin S\right\}$, are ignored in the feature construction.

### L_2_-regularized logistic regression for large data training and noise tolerance

The existing interlog modeling methods [22–25] have demonstrated two major disadvantages. First, they do not discriminate less significant interlogs from significant interlogs, less significant interlogs need to be further validated; second, they cannot detect those interactions that exist inexplicitly in the form of interlogs. To solve the two problems, we combine machine learning approach with interlog modeling via two-level knowledge transfer, namely interaction-level interlog knowledge transfer and protein−/gene-level homolog knowledge transfer. The former level of knowledge transfer is to derive the significant interlogs as the training data, and the second level of knowledge transfer is to make up for the sparsity of GO terms. The two-level knowledge transfer demands that the machine learning methods we choose should be resistant to noise. SVM support vector machine (SVM) [43] is a theoretically established machine learning method known for its regularization technique resistant to noise/outliers. Unfortunately, SVM is not applicable to large training data due to its time complexity *O*(*n*^2^). Here we adopt the l_2_-regularized logistic regression method [44], implemented in the toolbox LIBLINEAR [45], to counteract the ortholog/homolog noise and fit the large training data in linear time.

Given a set of instance-label pairs (*x*_*i*_, *y*_*i*_), *i* = 1, 2, …, *l*; *x*_*i*_ ∈ *R*^*n*^; *y*_*i*_ ∈ {−1, +1}, linear regression attempts to derive a decision function *f*(*x*_*i*_) = *ω*^*T*^*x*_*i*_ + *b*, which is further converted to probability via the logistic function *p*(*y* =  ± 1| *ω*, *x*_*i*_) = 1/1 + exp(−*y*_*i*_(*ω*^*T*^*x*_*i*_ + *b*)). The weight vector and bias (*ω*, *b*) could be estimated by minimizing the negative log-likelihood $\underset{\omega, b}{\min}\sum \limits_{i=1}^l\log \left(1+{e}^{-{y}_i\left({\omega}^T{x}_i+b\right)}\right)$. L_2_-regularized logistic regression imposes a constraint on the l_2_-norm of the weight vector *ω* to solve the following unconstrained optimization problem [44].

10 $$ \underset{\omega }{\min}\frac{1}{2}{\omega}^T\omega +C\sum \limits_{i=1}^l\log \left(1+{e}^{-{y}_i\left({\omega}^T{x}_i+\mathrm{b}\right)}\right) $$

where *C* denotes the penalty parameter/regularizer that balances the two terms in Formula (10) to achieve good generalization ability. The second term could penalize potential noise/outlier fitting. The optimization of objective function (10) is solved via its dual form.

11 $$ {\displaystyle \begin{array}{l}\underset{\alpha }{\min}\frac{1}{2}{\alpha}^T Q\alpha +\sum \limits_{i:{\alpha}_i>0}^l{\alpha}_i\log {\alpha}_i+\sum \limits_{i:{\alpha}_i<C}\left(C-{\alpha}_i\right)\log \left(C-{\alpha}_i\right)-\sum \limits_i^lC\log C\\ {} subjectto0\le {\alpha}_i\le C,i=1,\dots, l\end{array}} $$

where *α*_*i*_ denotes Lagrangian operator and ${Q}_{ij}={y}_i{y}_j{x}_i^T{x}_j$.

In the test and prediction phase, the decision function *f*(*x*) yields two outputs $f\left({V}_T^{\left({i}_1,{i}_2\right)}\right),f\left({V}_H^{\left({i}_1,{i}_2\right)}\right)$ for each protein-protein pair (*i*_1_, *i*_2_), which are further combined into one final decision as follows.

12 $$ F\left({V}_T^{\left({i}_1,{i}_2\right)},{V}_H^{\left({i}_1,{i}_2\right)}\right)=\left\{\begin{array}{l}f\left({V}_T^{\left({i}_1,{i}_2\right)}\right), if\mid f\left({V}_T^{\left({i}_1,{i}_2\right)}\right)\mid >\mid f\left({V}_H^{\left({i}_1,{i}_2\right)}\right)\mid \\ {}f\left({V}_H^{\left({i}_1,{i}_2\right)}\right), otherwise\end{array}\right. $$

where ∣Δ∣ denotes the absolute value of Δ. The final label for protein pair (*i*_1_, *i*_2_) is defined as below.

13 $$ L\left({i}_1,{i}_2\right)=\left\{\begin{array}{l}1, ifF\left({V}_T^{\left({i}_1,{i}_2\right)},{V}_H^{\left({i}_1,{i}_2\right)}\right)>\zeta \\ {}0, otherwise\end{array}\right. $$

The threshold *ζ* is used to filter out those weak positive predictions.

### Experimental setting and model evaluation

As described above, each protein pair (*i*_1_, *i*_2_) is represented with two instances, the target instance ${V}_T^{\left({i}_1,{i}_2\right)}$ and the homolog instance ${V}_H^{\left({i}_1,{i}_2\right)}$, so that the proposed framework yields three outputs for decision, i.e.$f\left({V}_T^{\left({i}_1,{i}_2\right)}\right)$, $f\left({V}_H^{\left({i}_1,{i}_2\right)}\right)$, $F\left({V}_T^{\left({i}_1,{i}_2\right)},{V}_H^{\left({i}_1,{i}_2\right)}\right)$, respectively. Accordingly, we design three experimental settings, namely combined-instance ($F\left({V}_T^{\left({i}_1,{i}_2\right)},{V}_H^{\left({i}_1,{i}_2\right)}\right)$), homolog-instance ($f\left({V}_H^{\left({i}_1,{i}_2\right)}\right)$) and target-instance ($f\left({V}_T^{\left({i}_1,{i}_2\right)}\right)$), to validate the effectiveness of homolog knowledge transfer. The combined-instance setting combines the outputs of the target instance and the homolog instance, the homolog-instance setting uses the homolog instance alone to evaluate the model robustness to GO term sparsity, and the target-instance setting uses the target instance alone to yield the baseline performance, equivalence to or excellence over which indicates that homolog knowledge transfer is effective.

Five performance metrics, i.e. ROC-AUC (Receiver Operating Characteristic AUC), SE (sensitivity), SP (specificity), MCC (Matthews correlation coefficient) and F1 score, are used to evaluate the proposed model via 5-fold cross validation. The dataset is randomly split into five disjoint parts. For five folds, each fold treats one part as test set and the other four parts are merged as training set. For each test example, the true label and the predicted label are recorded into the confusion matrix *M*. When the five folds complete, we use *M* to calculate the performance metrics. Except ROC-AUC, all the other metrics are derived from the confusion matrix *M*. From *M*, we define several intermediate variables as formula (14). Based on these intermediate variables, we further define SP_l_, SE_l_ and MCC_l_ for each label as formula (15) and the overall MCC as formula (16).

14 $$ {\displaystyle \begin{array}{l}{p}_l={M}_{l,l},{q}_l=\sum \limits_{i=1,i\ne l}^L\sum \limits_{j=1,j\ne l}^L{M}_{i,j},{r}_l=\sum \limits_{i=1,i\ne l}^L{M}_{i,l},{s}_l=\sum \limits_{j=1,j\ne l}^L{M}_{l,j}\\ {}p=\sum \limits_{l=1}^L{p}_l,q=\sum \limits_{l=1}^L{q}_l,r=\sum \limits_{l=1}^L{r}_l,s=\sum \limits_{l=1}^L{s}_l\end{array}} $$

15 $$ {\displaystyle \begin{array}{l}{SP}_l=\raisebox{1ex}{${p}_l$}\!\left/ \!\raisebox{-1ex}{${p}_l+{r}_l$}\right.,l=1,2\dots, L\\ {}{SE}_l=\raisebox{1ex}{${p}_l$}\!\left/ \!\raisebox{-1ex}{${p}_l+{s}_l$}\right.,l=1,2\dots, L\\ {}{MCC}_l=\left({p}_l{q}_l-{r}_l{s}_l\right)/\sqrt{\left({p}_l+{r}_l\right)\left({p}_l+{s}_l\right)\left({q}_l+{r}_l\right)\left({q}_l+{s}_l\right)},l=1,2\dots, L\end{array}} $$

16 $$ {\displaystyle \begin{array}{l} Acc={\sum}_{l=1}^L{M}_{l,l}/{\sum}_{i=1}^L{\sum}_{j=1}^L{M}_{i,j}\\ {} MCC=\left( pq- rs\right)/\sqrt{\left(p+r\right)\left(p+s\right)\left(q+r\right)\left(q+s\right)}\end{array}} $$

where the element of confusion matrix *M*_*i*, *j*_ records the counts that class *i* are classified to class *j*, and *L* denotes the number of labels. AUC is calculated based on the decision values defined in formula (12). F1 score is defined as follows:

17 $$ F1 score=\raisebox{1ex}{$2\times {SP}_l\times {SE}_l$}\!\left/ \!\raisebox{-1ex}{${SP}_l+{SE}_l$}\right.,l=1 denotesthepositiveclass $$

## Results

### Quality validation on the constructed data via GO enrichment analysis

The E-value cut-off for significant interlogs is set *δ* = 1e ‐ 50. To date, there is no commonly-accepted standard to choose PSI-Blast E-value cut-off. We are inclined to choose a small E-value cut-off to obtain quality interlogs. As results, we obtain 15,287 significant interlogs as the positive examples (see Additional file 1), 15,287 randomly sampled non-interlogs as the negative examples (see Additional file 2), a set containing 98,187 less significant interlogs, a set containing 98,187 non-interlogs that are potentially negative examples, and a prediction set containing 1359,00 protein pairs. The sampling ratio of negative examples to positive examples is set 1:1 for the two reasons: (1) skewed distributions between the positive class and the negative class (e.g. ratio 1:10, 1:100, etc.) could increase the risk of model bias; (2) there is actually no direct mapping from the biological problem space to the computational space, so it is improper to simulate the huge negative space by sampling a much larger negative data set to train a predictive model from a computational point of view.

**Fig. 2:**
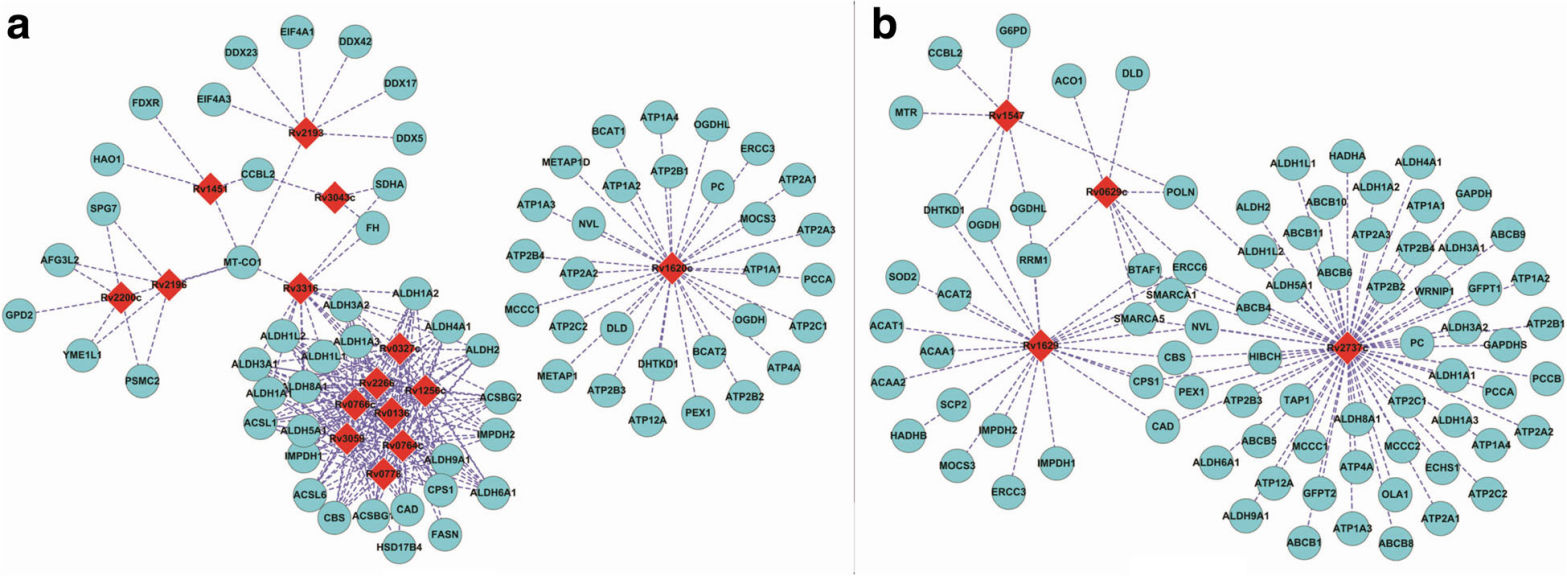
The derived interlogs between *M. tuberculosis H37Rv* drug-resistant genes and human genes. **a** illustrates the interactions of *M. tuberculosis* H37Rv drug-resistant genes involved in cytochromes and other target-modifying enzymes that could cause potential chemical modification of drug molecules [46]. **b** illustrates the interactions of *M. tuberculosis* H37Rv drug-resistant genes involved in SOS-response and DNA replication that lead to mutations in the gene or its regulatory region. The light blue circles denote *M. tuberculosis* H37Rv genes and the red diamonds denote human genes [46]

The significant interlogs are directly viewed as MTB-human protein interactions, so we need to assess the quality of the derived interlogs and their potential applications. As there is no independent benchmark measure, we analyse the quality of interlogs only from the aspects of similar GO terms. Of course, it is the feature vector of GO terms as Formula (9) defines that determines the predictive output. In addition, we analyse the drug resistance related interlogs to reveal the role of the host factors in the progression of bacterial antibiotic resistance. The less significant interlogs need to be further validated by the proposed framework and will be analysed in the following subsection. Antimicrobial peptides (AMPs) represent a potential alternative to available antibiotics. Raman et al. [46] exploit the *M. tuberculosis* H37R PPI networks to find so-called co-target genes whose co-inhibition with the resistance genes could effectively blockade *M. tuberculosis* H37RV signaling pathways. Nevertheless, inhibitors of pathogen-host PPI interface could be more therapeutically specific to bacterial infection with less risk of drug resistance and drug side-effects. Figure 2a illustrates the significant interlogs related to *M. tuberculosis* H37Rv drug-resistant genes that get involved in cytochromes and other target-modifying enzymes [46], which could cause potential chemical modification of drug molecules (see Additional file 3 for detailed GO enrichment analysis). Figure 2b illustrates the significant interlogs related to *M. tuberculosis* H37Rv drug-resistant genes that get involved in SOS-response and DNA replication [46], which could lead to mutations in the gene or its regulatory region (see Additional file 4 for detailed GO enrichment analysis).

#### Interlog {Rv2193, MT-CO1}

The drug-resistant gene *Rv2193* is involved in cytochromes and other target-modifying enzymes that could cause potential chemical modification of drug molecules [46]. The interlog {*Rv2193*|I6Y8N5, *MT-CO1|*P003951} is inferred from the known *M. tuberculosis* H37Rv protein interaction {*Rv2193*|I6Y8N5, *Rv3043c*|I6YAZ7} [7], where the human protein P003951 (*MT-CO1*) is orthologous to the *M. tuberculosis* H37Rv protein I6YAZ7 (*Rv3043c*) with E-value equal to 4e-094. From GO enrichment analysis as partially provided in Table 1, the two genes {*Rv2193*, *MT-CO1*} both get involved in the common biological processes of oxidation-reduction process (GO:0055114) and hydrogen ion transmembrane transport (GO:1902600). In addition, the two genes are also both involved in aerobic cellular respiration, e.g. *Rv2193* respiratory electron transport chain (GO:0022904) and *MT-CO1* aerobic respiration (GO:0009060). Besides, the two orthologous proteins {*Rv3043c*|I6YAZ7, *MT-CO1|*P003951} are also involved in other highly similar biological processes (see Additional file 3).

**Table 1:** GO enrichment analysis of the significant interlog {*Rv2193*, *MT-CO1*} *LH57_11955* (*Rv2193*) is classified into the drug resistance type of cytochromes and other target-modifying enzymes that could cause potential chemical modification of drug molecules [46]

	GO term ID	GO aspect	GO term name
Common GO terms	GO:0016020	C	membrane
	GO:0016021	C	integral to membrane
	GO:0016491	F	oxidoreductase activity
	GO:0055114	P	oxidation-reduction process
	GO:1902600	P	hydrogen ion transmembrane transport
*H57_11955*|Rv2193 only	GO:0022904	P	respiratory electron transport chain
	GO:0019646	P	aerobic electron transport chain
	GO:0015002	F	heme-copper terminal oxidase activity
*MT-CO1* only	GO:0070469	C	respiratory chain
	GO:0045277	C	respiratory chain complex IV
	GO:0005751	C	mitochondrial respiratory chain complex IV
	GO:0006979	P	response to oxidative stress
	GO:0009060	P	aerobic respiration
	GO:0046688	P	response to copper ion
	GO:0051602	P	response to electrical stimulus
	GO:0020037	F	heme binding

C denotes cellular component, F denotes molecular function, and P denotes biological process

#### Interlog {Rv2737c, ERCC6}

The drug-resistant gene *Rv2737c* is involved in SOS-response and DNA replication that lead to mutations in the gene or its regulatory region. The interlog {*Rv2737c*|I6YE90, *ERCC6|*Q03468} is inferred from the known *M. tuberculosis* H37Rv protein interaction {*Rv2737c*|I6YE90, *helZ|Rv2101|*I6YCF3} [7], where the human protein Q03468 (*ERCC6*) is orthologous to the *M. tuberculosis* H37Rv protein I6YCF3 (*Rv2101*, gene name *helZ*) with E-value equal to 5e-058. The GO enrichment analysis of the two genes {*Rv2737c*, *ERCC6*} is partially provided in Table 2. We can see that these two genes get involved in the common biological processes of DNA repair (GO:0006281) and response to DNA damage stimulus (GO:0006974). Besides, the gene *Rv2737c* protects microbial DNA from antibiotics (GO:0046677, response to antibiotic), DNA damage (GO:0009432, SOS response), ultraviolet radiation (GO:0009650, UV protection), ionizing radiation (GO:0010212, response to ionizing radiation), etc. Accordingly, the human gene *ERCC6* gets involved in response to gamma radiation (GO:0010332), response to UV (GO:0009411), response to oxidative stress (GO:0006979), DNA damage response, signal transduction resulting in induction of apoptosis (GO:0008630), base-excision repair (GO:0006284), etc. The similar biological processes suggest that the two genes {*Rv2737c*, *ERCC6*} potentially interact.

**Table 2:** GO enrichment analysis of the derived interlog {*Rv2737c*, *ERCC6*}. *recA* (*Rv2737c*) is classified into the drug resistance type of SOS-response and DNA replication that leads to mutations in the gene or its regulatory region [46]

	GO term ID	GO aspect	GO term name
Common GO terms	GO:0005515	F	protein binding
	GO:0006281	P	DNA repair
	GO:0006974	P	response to DNA damage stimulus
	GO:0016787	F	hydrolase activity
	GO:0008094	F	DNA-dependent ATPase activity
	GO:0003677	F	DNA binding
*recA*|Rv2737c only	GO:0046677	P	response to antibiotic
	GO:0009432	P	SOS response
	GO:0009650	P	UV protection
	GO:0010212	P	response to ionizing radiation
	GO:0006310	P	DNA recombination
	GO:0006259	P	DNA metabolic process
	GO:0000725	P	recombinational repair
*ERCC6* only	GO:0000303	P	response to superoxide
	GO:0006283	P	transcription-coupled nucleotide-excision repair
	GO:0006284	P	base-excision repair
	GO:0006979	P	response to oxidative stress
	GO:0007256	P	activation of JNKK activity
	GO:0008630	P	DNA damage response, signal transduction resulting in induction of apoptosis
	GO:0010332	P	response to gamma radiation
	GO:0009411	P	response to UV

C denotes cellular component, F denotes molecular function, and P denotes biological process

### Performance of 5-fold cross validation

**Fig. 3:**
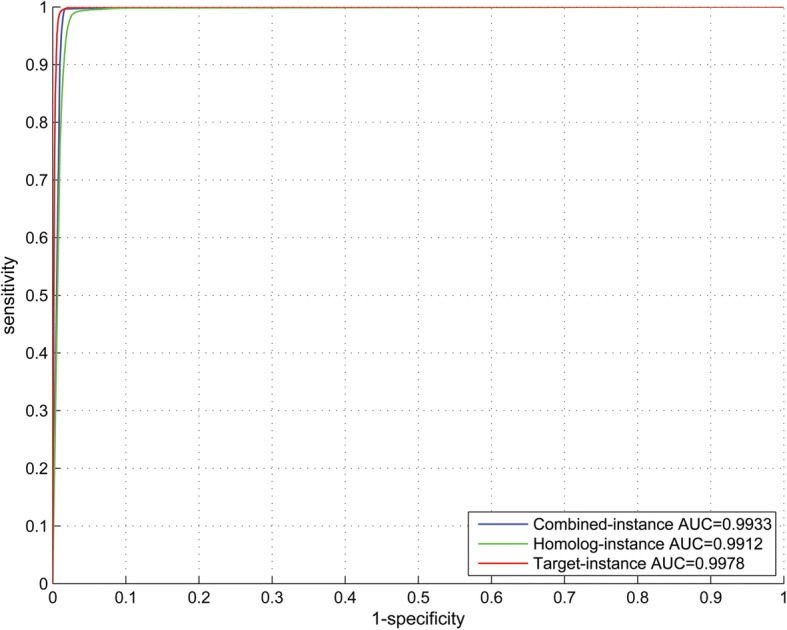
ROC curves for 5-fold cross validation performance evaluation on the artificially created significant interlogs between *M. tuberculosis* H37Rv and *H. sapiens*

The ROC curves of 5-fold cross validation are illustrated in Fig. 3 and the detailed performance metrics are provided in Table 3. The proposed method achieves fairly good performance in terms of all the performance measures, and the performance varies very little between the three experimental settings, which indicate that the homolog instances are effective to substitute the target instances when the GO knowledge of the genes concerned is not available.

**Table 3:** Performance estimation of 5-fold cross validation and performance comparison with the existing methods

	Size	Combined-instance			Homolog-instance			Target-instance		
		SP	SE	MCC	SP	SE	MCC	SP	SE	MCC
Positive	15,287	0.9823	0.9966	0.9790	0.9684	0.9915	0.9601	0.9820	0.9976	0.9796
Negative	15,287	0.9965	0.9821	0.9790	0.9912	0.9676	0.9601	0.9975	0.9817	0.9796
[Acc; MCC]		[98.93%; 0.9789]			[97.95%; 0.9599]			[97.95%; 0.9599]		
[ROC-AUC]		[0.9933]			[0.9912]			[0.9978]		
F1 Score		0.9894			0.9798			0.9897		
KMM-SVM [23]		SP			SE			F1 score		
① human- > mouse		0.517			0.937			0.667		
② *E.coli*- > *human*		0.257			0.161			0.199		

① denotes the work [23] that transfers the knowledge of *Salmonella*-human PPI networks to predict *Salmonella*-mouse protein interactions;② denotes the work [23] that transfers the knowledge of *Salmonella*-*Ecoli* PPI networks to predict *Salmonella*-human protein interactions

Furthermore, the proposed method achieves quite well-balanced performance on the two classes, indicating that the positive class of significant interlogs and the negative class of non-interlogs are well separated. In the section *Analysis of the constructed data*, we have analysed the quality of the derived significant interlogs via GO enrichment analysis. To well interpret the good two-class separability, we conduct further GO enrichment analysis on the positive and the negative training data.

**Fig. 4:**
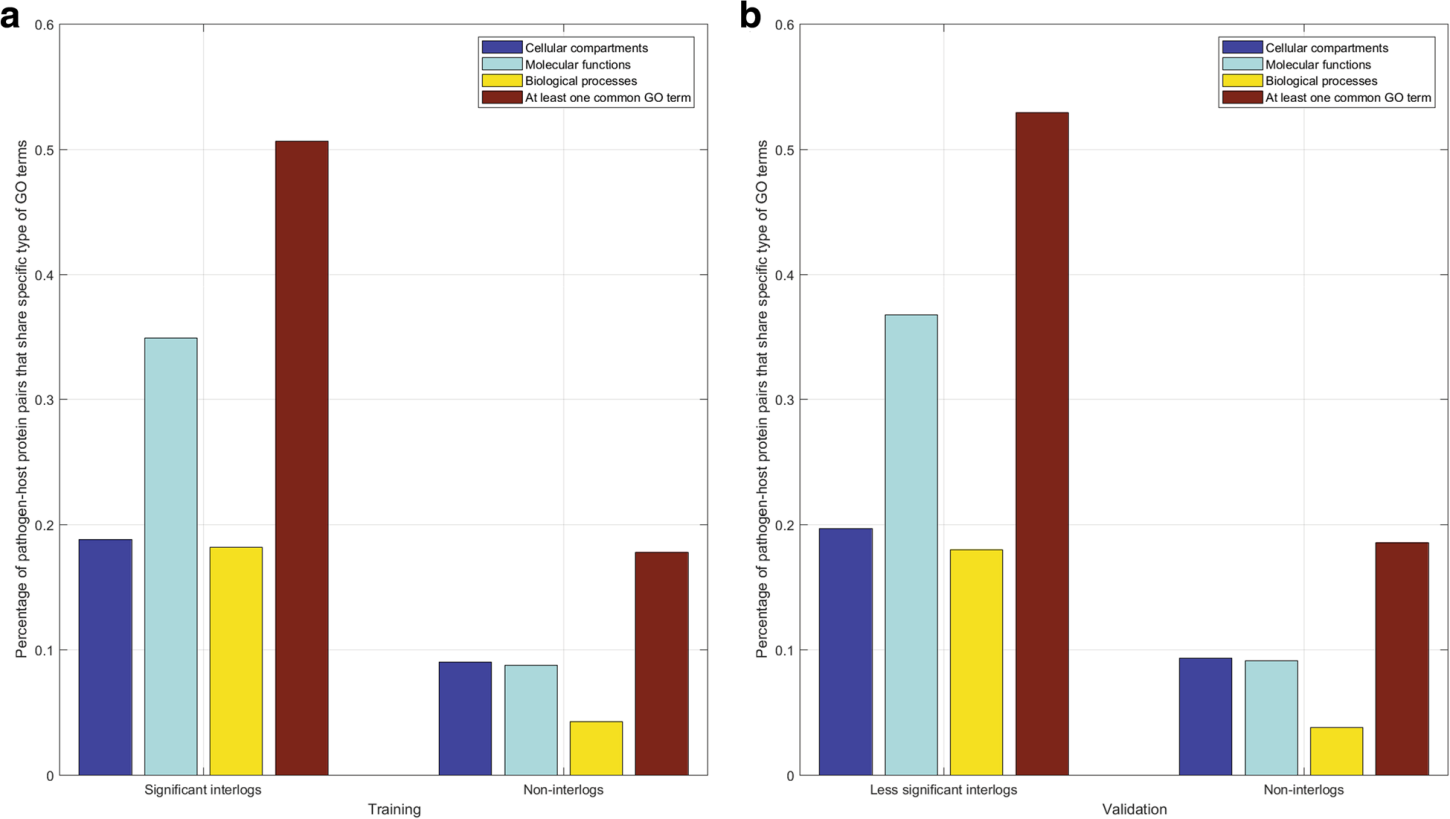
Percentage of pathogen-host protein pairs in the training data whose partners share common GO terms

As illustrated in Fig. 4a, the protein pairs in the positive training data (i.e. significant interlogs) show more significant common patterns of subcellular localization, molecular functionality and biological processes than those in the negative training data (i.e. non-interlogs). Such a wide difference of common patterns between the positive data and the negative data presumably contribute much to the two-class separability, which then results in the good performance of 5-fold cross validation. The results as illustrated in Fig. 4a are consistent with the observations that two proteins that interact are more likely to reside in the same cellular compartments, fulfil similar molecular functions and participate in similar biological processes. As mentioned in the section Background, the complex bacterial cell wall that forms a strong permeability barrier to the mutual access of the bacterial genome and the host genome [19], the two partners of significant interlogs may merely functionally interact if no transport or secretion helps the two partners physically contact.

### Quality validation on the predicted interactions from less significant interlogs and non-interlogs

At present there is no experimental data available as validation set to evaluate the proposed model. Nevertheless, the less significant interlogs would be more likely to be interacting partners than non-interlogs. For the reasons, we explicitly study the potential interactions from the less significant interlogs and the non-interlogs, partly to evaluate the proposed model as well. The predicted interactions from the less significant interlogs and the non-interlogs are provided in Additional files 5 and 6, respectively. As shown in Table 4, the predicted positive rates on the less significant interlogs and non-interlogs are 18.78 and 1.41%, respectively. We can see that the less significant interlogs are more likely to interact than the non-interlogs. The result can be well interpreted by the wide difference of patterns of common GO terms between the less significant interlogs and the non-interlogs as illustrated in Fig. 4b. Comparing Fig. 4a with Fig. 4b, we see that the interlogs show much are similar distributions of GO terms than non-interlogs. The low positive rate 18.78% indicates that the less significant interlogs should not be equally treated as the significant interlogs as the existing work does [22–25], and need to be further validated by a machine learning framework. The low positive rate 1.41% shows that the sampling method of negative data as described in Formula (4) is rational.

**Table 4:** Predicted positive rates on less significant interlogs, non-interlogs and the prediction set

	Less significant interlogs	Non-interlogs	Prediction set
Size	98,187	98,187	407,700
Predicted positive rate	18.78%	1.41%	1.96%

**Fig. 5:**
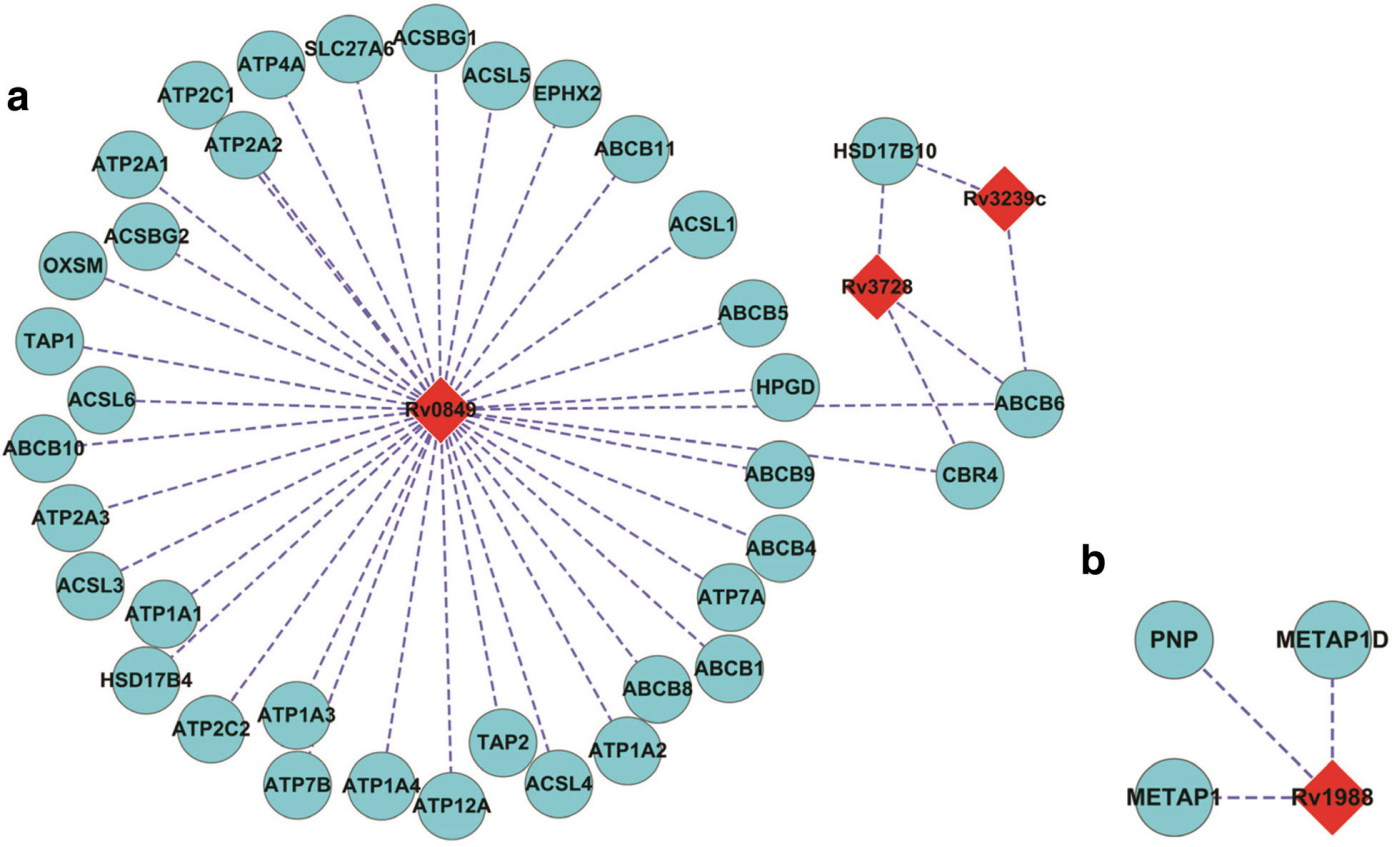
Validated less significant interlogs. **a** illustrates the interactions of *M. tuberculosis* H37Rv drug-resistant genes that are involved in antibiotic efflux pumps [46]. **b** illustrates the interactions of *M. tuberculosis* H37Rv drug-resistant genes that are involved in target-modifying enzymes [46]

As reviewed in [19], host factors play important roles in the progression of bacterial drug resistance. Hence the interactions between *M. tuberculosis* H37Rv drug-resistant genes and human host genes are of special interest to us. Moreover, inhibitors of pathogen-host PPI interface would be more therapeutically with less side-effect on other human genes and pathways. The predicted interactions from less significant interlogs related to drug resistance are illustrated in Fig. 5. Figure 5a illustrates the interlogs that get involved in antibiotic efflux pumps [46] (see Additional file 7 for detailed GO enrichment analysis), and Fig. 5b illustrates the interlogs that get involved in target-modifying enzymes [46] (see Additional file 8 for detailed GO enrichment analysis).

#### Interlog {Rv0849, ABCB1}

The interlog {*Rv0849|*I6X9Y5*, ABCB1|*P08183} is derived from the known interaction {*Rv0849|*I6X9Y5, *Rv1348|*I6YAB3} [7], wherein the human protein P08183is orthologous to the *M. tuberculosis* H37Rv protein I6X9Y5 with E-value equal to 6e-043. The interlog {*Rv0849|*I6X9Y5*, ABCB1|*P08183} is predicted to be a pathogen-host protein interaction with probability 0.9987 (see Additional file 7). GO enrichment analysis shows that the two genes {*Rv0849, ABCB1*} both are located at membrane (GO:0005886, plasma membrane; GO:0016021, integral to membrane) and participate the biological process of transport (GO:0055085, transmembrane transport; GO:0006810, transport) (see Table 5). In addition, the human gene ABCB1 also gets involved in the biological processes of drug transmembrane transport (GO:0006855) and xenobiotic transport (GO:0042908). The GO terms (GO:0009986, cell surface; GO:0070062, extracellular vesicular exosome) indicate that the human protein P08183 could have physical contact with the *M. tuberculosis* H37Rv membrane protein I6X9Y5 to induce immune response (GO:0002485, antigen processing and presentation of endogenous peptide antigen via MHC class I via ER pathway, TAP-dependent).

**Table 5:** Gene ontology analysis of the predicted interactions {*Rv0849*, *ABCB1*} and {*Rv1988*, *PNP*}. *Rv0849* is classified into the drug resistance type of antibiotic efflux pumps [46]. *Rv1988* is classified into the drug resistance type of target-modifying enzymes [46]

{*Rv0849, ABCB1*}	GO term ID	GO aspect	GO term name
Common GO terms	GO:0055085	P	transmembrane transport
	GO:0006810	P	transport
	GO:0005886	C	plasma membrane
	GO:0016021	C	integral to membrane
*ABCB1* only	GO:0005215	F	transporter activity
	GO:0006855	P	drug transmembrane transport
	GO:0042908	P	xenobiotic transport
	GO:0009986	C	cell surface
	GO:0070062	C	extracellular vesicular exosome
	GO:0002485	P	antigen processing and presentation of endogenous peptide antigen via MHC class I via ER pathway, TAP-dependent
{*Rv1988*, *PNP*}	GO term ID	GO aspect	GO term name
Common GO terms	GO:0005737	C	cytoplasm
	GO:0016740	F	transferase activity
*Rv1988* only	GO:0000154	P	rRNA modification
	GO:0008649	F	rRNA methyltransferase activity
	GO:0031167	P	rRNA methylation
	GO:0046677	P	response to antibiotic
*PNP only*	GO:0006139	P	nucleobase-containing compound metabolic process
	GO:0006148	P	inosine catabolic process
	GO:0006195	P	purine nucleotide catabolic process
	O:0006738	P	nicotinamide riboside catabolic process
	GO:0006955	P	immune response
	GO:0042493	P	response to drug
	GO:0070970	P	interleukin-2 secretion
	GO:0034356	P	NAD biosynthesis via nicotinamide riboside salvage pathway

C denotes cellular component, F denotes molecular function, and P denotes biological process

#### Interlog {Rv1988, PNP}

The interlog {*Rv1988|*Q10838*, PNP*|P00491} is derived from the known interaction {*Rv1988|Q10838*, *Rv0535|*I6Y409} [7]. The human protein P00491 is orthologous to the *M. tuberculosis* H37Rv protein I6Y409 with E-value equal to 6e-034. The *M. tuberculosis* H37Rv gene *Rv1988* is classified into the drug resistance type of target-modifying enzymes [46]. As shown in Table 5, the *M. tuberculosis* H37Rv *Rv1988* gets involved in the biological processes of rRNA modification (GO:0000154), rRNA methylation (GO:0031167) and response to antibiotic (GO:0046677), while the human gene *PNP* also gets involved in the biological processes of protein modifications, e.g. the catabolic processes of nucleobase-containing compound (GO:0006139), inosine (GO:0006148), purine nucleotide (GO:0006195), etc. In addition, *PNP* is involved in the biological processes of immune response (GO:0006955, GO:0070970) and response to drug (GO:0042493).

### Predicted interactions on the prediction set

The prediction set that contains 407,700 MTB-human protein pairs is derived from the huge space of non-interlogs according to Formula (7). As shown in Tables 1, 4.96% of protein pairs are predicted to be pathogen-host PPIs. Such a low positive rate is presumably rational with a low risk of false positive predictions. The predicted interactions on the prediction set are provided in Additional file 9. For the convenience of analysis, we merge the significant interlogs together with the predicted interactions from the less significant interlogs, non-interlogs and the prediction set into Additional file 10. We totally obtain 43,116 predicted protein interactions between *M. tuberculosis* H37Rv and *Homo sapiens*, which is still incomplete since the prediction set is only a small part of the prediction space.

**Fig. 6:**
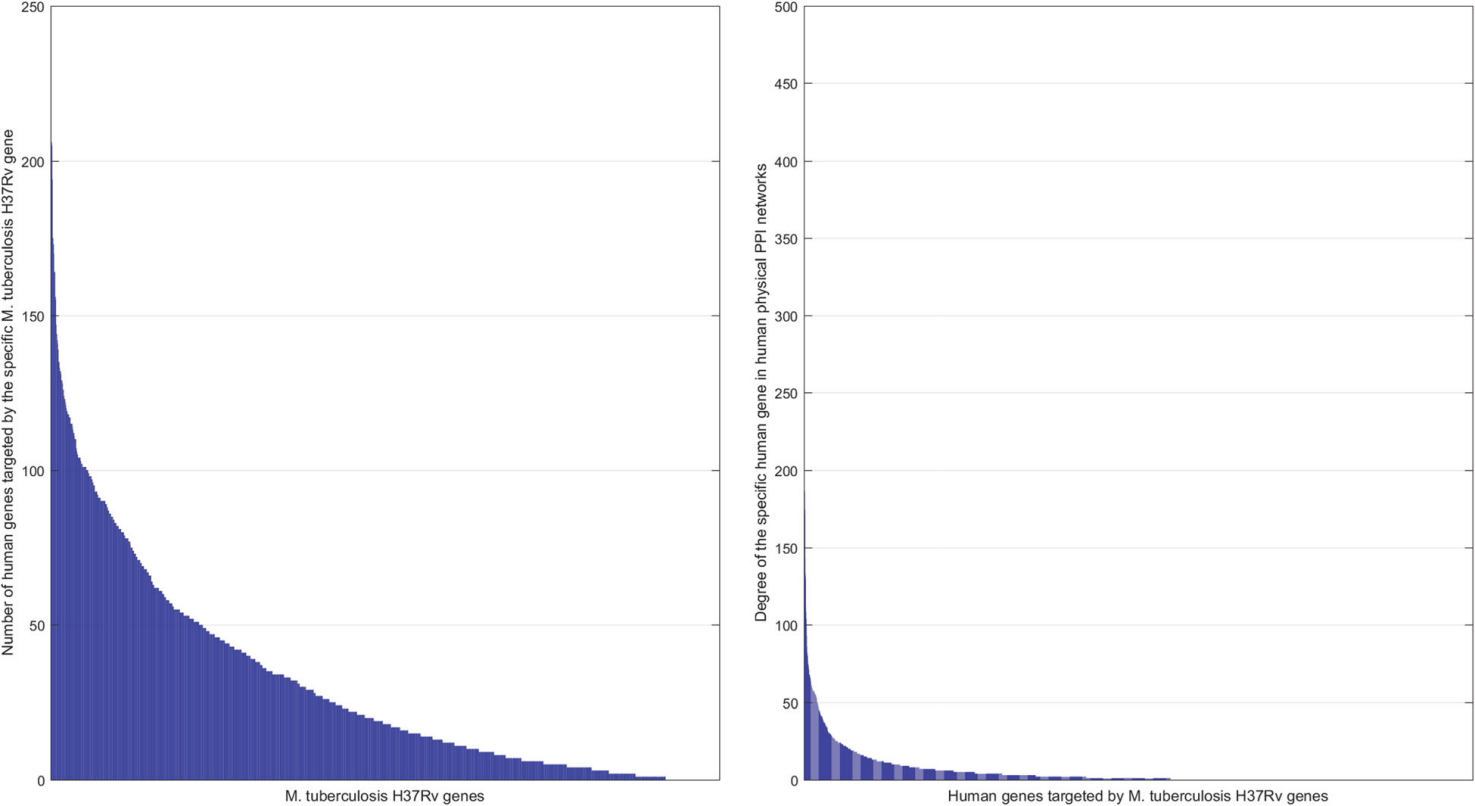
The degree distribution of the *M. tuberculosis* H37Rv genes in the derived MTB-human PPI networks (left); the degree distribution of *M. tuberculosis* H37Rv targeted human genes in human physical PPI networks (right)

Taking advantage of the predicted MTB-human PPI networks, we need to address two concerns (1) how many human genes a *M. tuberculosis* H37Rv gene is likely to target; (2) what roles the targeted human genes play in the human PPI networks. The two concerns are actually about two kinds of degree distributions (1) the degree distribution of the *M. tuberculosis* H37Rv genes in the MTB-human PPI networks (see Fig. 6 (left)); (2) the degree distribution of the human genes in human PPI networks (see Fig. 6 (right)). We can see that the two degrees show a tendency of power-law distribution. As shown in Fig. 6 (left), only a small portion of *M. tuberculosis* H37Rv genes are densely connected by human genes, indicating that only a small number of *M. tuberculosis* H37Rv genes intensively target dozens to several hundred of human genes. For instance, *M. tuberculosis* H37Rv gene *Rv0440* (*groEL*) and *Rv1436* (*LH57_07850*) interact with 209 and 206 human genes, respectively. As shown in Fig. 6 (right), only a small of targeted human genes are highly-connected hub genes and the long tail indicate that many targeted human genes are orphan genes in human PPI networks. It could be concluded that only a small number of *M. tuberculosis* H37Rv genes target a small number of human hub genes. The human PPI networks are constructed from HPRD [47] and BioGRID [48]. To further reveal the signaling cross-talks between *M. tuberculosis* H37Rv and *Homo sapiens*, we will discuss the patterns of *M. tuberculosis* H37Rv genes manipulating the human immune signaling pathways in the section *Discussions*.

## Discussions

To date, there are very few experimental studies on protein interactions between bacterial pathogens and their hosts. The latest database STRING has curated 1678 bacterial pathogen protein-protein interaction networks, but no work has been reported to exploit these networks to predict pathogen-host protein interactions thus far. Pathogen-host protein interactions play a critical role of signaling cross-talks between pathogen PPI networks and host PPI networks, which is of significance to understand the underlying mechanism of bacterial invasive infection and host immune response.

*Mycobacterium tuberculosis* is an obligate pathogenic bacterial species in the family of Mycobacteriaceae and the causative agent of tuberculosis. The physiology of *M. tuberculosis* is highly aerobic and requires high levels of oxygen. As primarily a pathogen of the mammalian respiratory system, *M. tuberculosis* mainly infects the lungs as well as other tissues. *M. tuberculosis* H37Rv has received much attention in recent years partly due to its co-infection with HIV and increasingly serious drug resistance. To date, the cross-talks or interactions between *M. tuberculosis* and *H. sapiens* proteins are much less understood than the individual genome of *M. tuberculosis*. To the best of our knowledge, there is no experimental study on protein interactions between *M. tuberculosis* H37Rv and *Homo sapiens*.

### Methodology comparison with the related computational methods

**Fig. 7:**
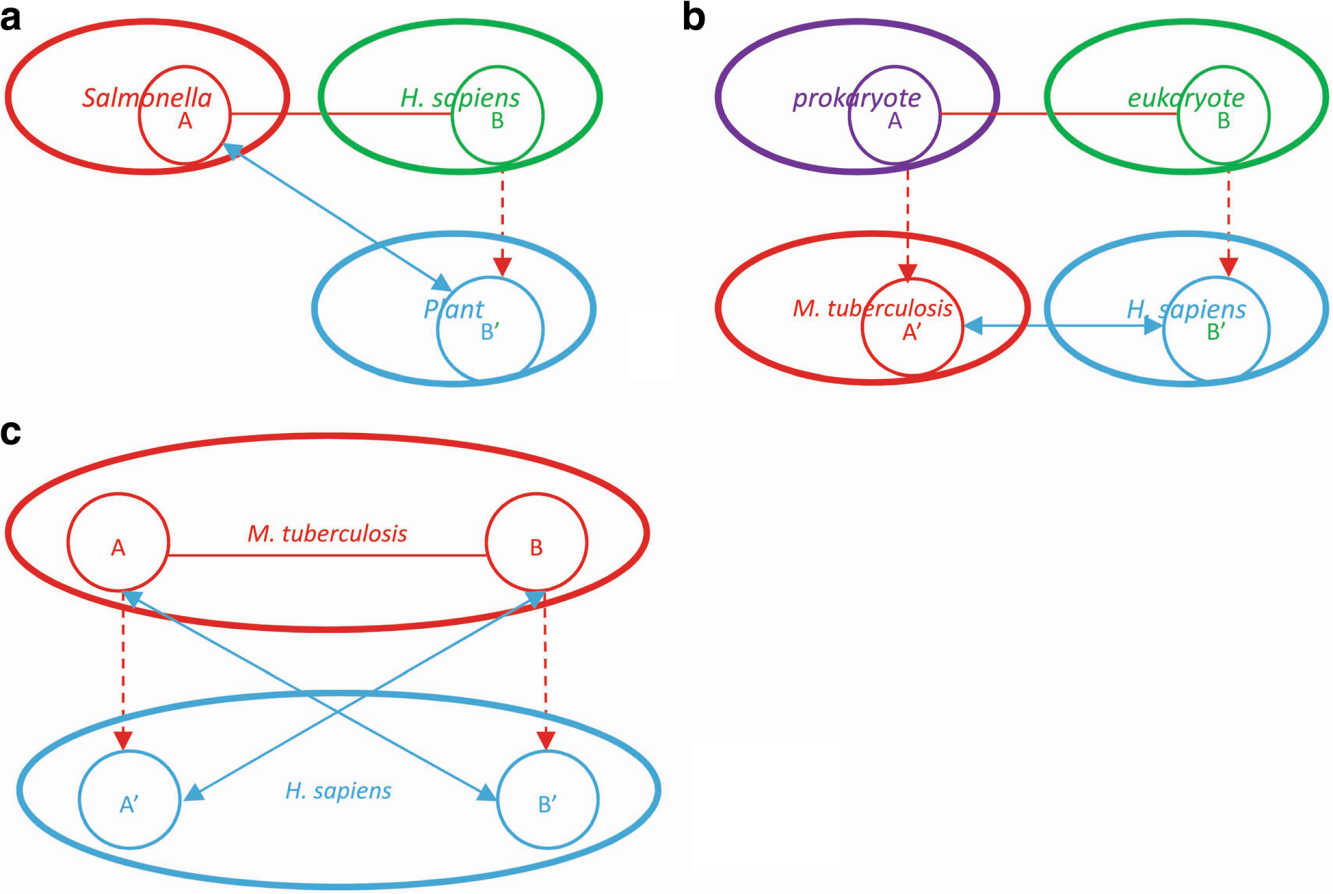
Illustration of the way of deriving interlogs. The ellipses in red and blue denote the target species and the ellipses in other colors denote the source species. The circles denote genes. The red full line denotes the experimental protein-protein interactions. The red dotted line denotes ortholog mapping. The blue full line with arrows at two ends denotes the derived interlogs. The methods illustrated in (**a**) and (**b**) exploited the pathogen-host PPIs of other species to derive the interlogs of the target species, while the method illustrated in (**c**) transferred the knowledge of intra-species *M. tuberculosis* H37Rv PPI networks to predict protein interactions between *M. tuberculosis* H37Rv and *H. sapiens*

The related computational methods generally infer interlogs via one or more third-party species. For instances, Kshirsagar et al. [23] use the known *Salmonella*-human PPIs as templates to infer *Salmonella*-plant PPIs via plant-human ortholog mapping (see Fig. 7a). Zhou et al. [24, 25] use the known prokaryote-eukaryote PPIs as templates to infer *M. tuberculosis*-*H. sapiens* PPIs via the ortholog mappings of prokaryote-*M. tuberculosis* and eukaryote-*H. sapiens* (see Fig. 7b). However, the large gap between the source species (e.g. plant) and the target species (e.g. human) is to a large extent prone to yield false pathogen-host protein interactions. In addition, the interlog-only methods [24, 25] neither validate the less significant interlogs nor train a predictive model to predict the non-interlogs that also potentially interact. Similar to the methods that combine interlog with machine learning approach [9, 23], we also use the derived interlogs as training data since there are no experimental data available, but differently we confine the search of *M. tuberculosis* H37Rv ortholog genes within the human host without resorting to a third-party species, meanwhile we do not need prior pathogen-host PPIs of other species as templates (see Fig. 7c). As illustrated in Fig. 7c, the ortholog genes of the two interacting *M. tuberculosis* H37Rv genes (*A*, *B*) are searched within the human genome space, presumably *A’* and *B′*, respectively, then it is assumed that *A* interacts with *B′* and *B* interacts with *A’*. The assumption is based on the accumulated evidences that the different strains of the obligate human pathogen *M. tuberculosi*s have co-evolved, migrated, and expanded with their human hosts [27]. Knowledge transfer between co-evolving species is more credible than that between evolutionarily distant species.

It is noted that the quality of *M. tuberculosis* H37Rv protein interaction networks in STRING [9] directly affects the quality of inferred interlogs between *M. tuberculosis* H37Rv and *Homo sapiens*. Among the *experimental* data in STRING [9], only 32 MTB PPIs are actually derived by experiments. Obviously, such a small data size cannot satisfy our needs, so we resort to the other *experimental* data in STRING [9] that are actually interlogs inferred from other experimentally-verified PPIs. Yu et al. [49] have testified the feasibility of interolog mapping, i.e. the transfer of interaction annotation from one organism to another using comparative genomics.

### Performance comparison with the related computational methods

The interlog-only methods [24, 25] do not provide baseline performance for comparison. The method that combines interlog with machine learning approach [23] derives *Salmonella*-plant interlogs from the known plant-human PPIs as the training data to train a KMM-SVM model for novel *Salmonella*-plant PPI predictions. As shown in Table 3, the knowledge of *Salmonella*-human PPI networks is transferred in the first experiment to predict *Salmonella*-mouse protein interactions, achieving SE 0.937 and SP 0.517; and the knowledge of S*almonella*-*Ecoli* PPI networks is transferred in the second experiment to predict *Salmonella*-human protein interactions, achieving SE 0.257 and SP 0.161. The results in the second experiment are obviously much poorer in that the gap of species between *E.coli* and human is much larger than that between mice and human. Even in the first experiment, the method [23] neither achieves satisfactory performance partly due to the other two reasons: (1) the less significant interlogs are not explicitly excluded out of the positive training data; (2) the two-class skew distribution also contributes to the low performance (SP = 0.517). In this work, the knowledge is only transferred across co-evolving pathogen and host, so that the proposed method achieves much better performance (see Table 3). Nevertheless, the proposed method also yields a certain level of bias and performance overestimation for the two reasons (1) similar interlogs in the training data could overestimate the performance of 5-fold cross validation performance, though they do not affect the final trained model and predictions; (2) the positive training data do not contain non-interlogs that also potentially interact because there are no such experimental data, so that the two classes of training data are easily separated. How to choose the representative interlogs to more objectively evaluate the proposed model is worth further consideration in the future work.

### GO enrichment analysis of the targeted human genes

**Fig. 8:**
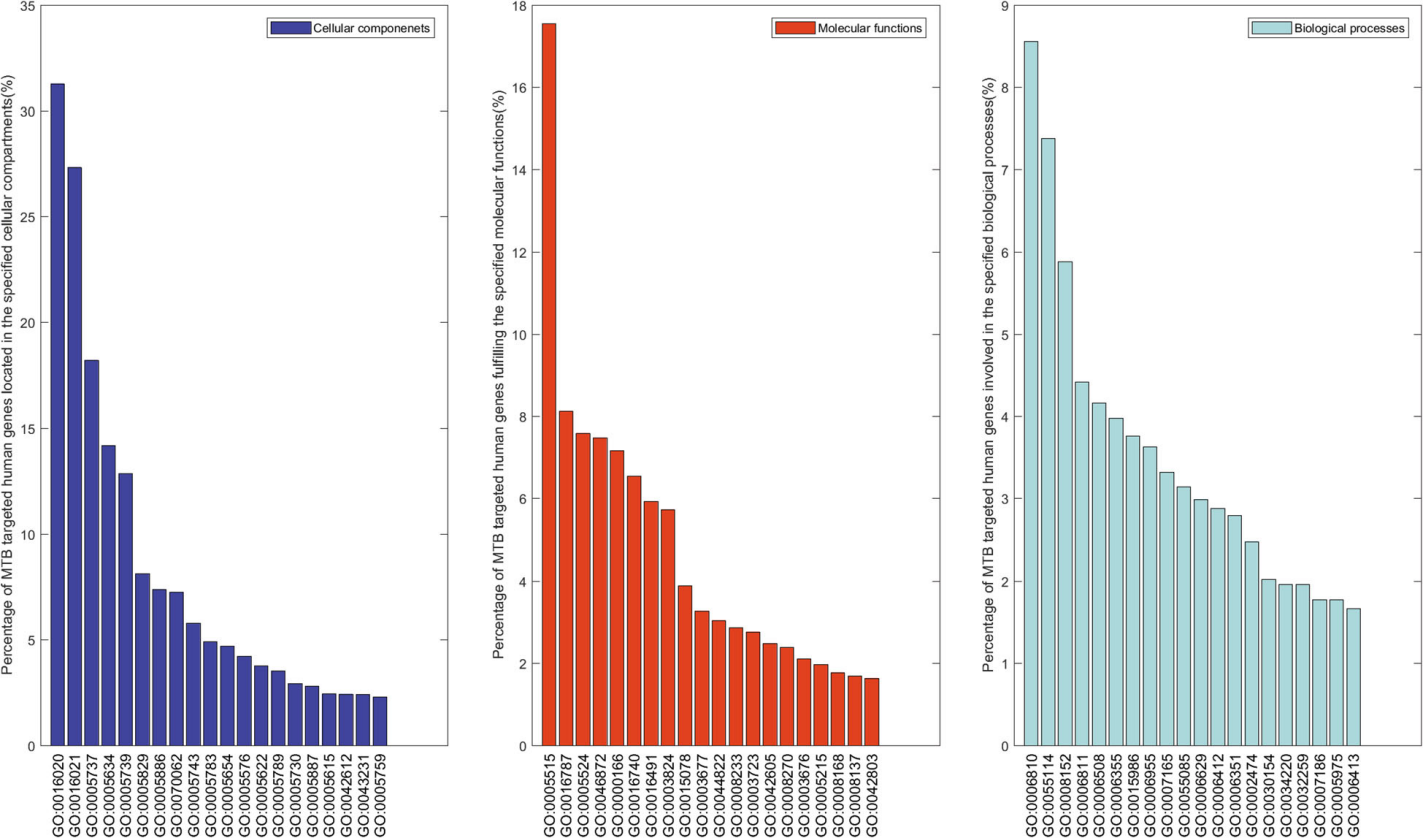
GO enrichment analysis of the human genes that are predicted to be targeted by *M. tuberculosis* H37Rv drug-resistant genes

Figure 8 illustrates the top 20 GO terms of human genes that are predicted to be targeted by *M. tuberculosis* H37Rv genes. As shown in Fig. 8 (left), *M. tuberculosis* H37Rv genes are inclined to target those human genes located in the cellular compartments of membrane (GO:0016020), integral to membrane (GO:0016021), cytoplasm (GO:0005737), nucleus (GO:0005634), mitochondrion (GO:0005739), etc. As shown in Fig. 8 (middle), the targeted human genes fulfil the molecular functions of protein binding (GO:0005515), hydrolase activity (GO:0016787), ATP binding (GO:0005524), metal ion binding (GO:0046872), oxidoreductase activity (GO:0016491), etc. As shown in Fig. 8 (right), the targeted human genes get involved in the biological processes of transport (GO:0006810), oxidation-reduction process (GO:0055114), metabolic process (GO:0008152), ion transport (GO:0006811), proteolysis (GO:0006508), regulation of transcription, DNA-dependent (GO:0006355), etc.

### Pathway enrichment analysis of the targeted human genes

**Fig. 9:**
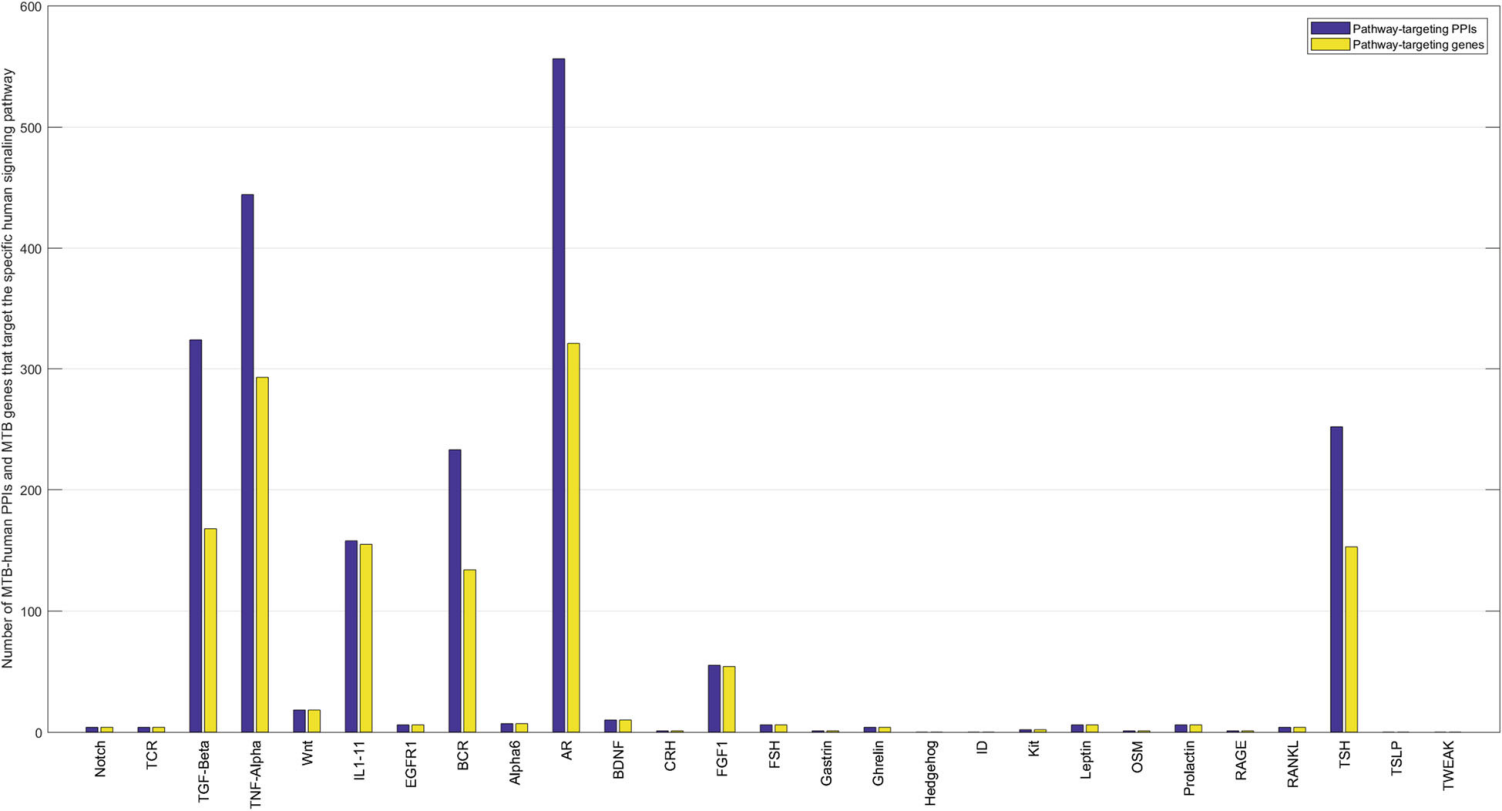
Statistics of MTB-human PPIs and MTB genes that are predicted to target specific human immune signaling pathways

Bacterial invasion could induce host inflammatory response, for instances, TNF-α is thought to play a role in the activation of resting macrophages and inhibition of bacterial dissemination, and IL-10 might play a role in controlling the trade-off between the anti-microbial activity and host-derived tissue caseation [50, 51]. We map the targeted human genes onto the known human immune signaling pathways curated in NetPath [52] to study how *M. tuberculosis* genes interact with human defence system. For simplicity, the pathways IL1~IL11 in NetPath are merged into one IL signaling pathway, thus we totally obtain 27 human immune signaling pathways. The predicted MTB-human PPIs related to human immune signaling pathways are provided in Additional file 11. As shown in Fig. 9, *M. tuberculosis* H37Rv genes are inclined to target the human immune signaling pathways of AR (Androgen receptor), TNF-alpha (Tumor necrosis factor alpha), TGF-beta (Transforming growth factor beta receptor), IL (Interleukin), BCR (B cell receptor), TSH (Thymic stromal lymphopoietin),, etc. In most cases, many *M. tuberculosis* H37Rv genes are predicted to target more than one human immune signaling pathways (see Additional file 12), for instances, *M. tuberculosis* H37Rv gene *recA* (Rv2737c) is predicted to target five signaling pathways (TNF-alpha;IL2;BCR;AR;TSH). Partial GO enrichment analysis of the targeted human genes on TNF-alpha and IL signaling pathways are given in Table 6.

**Table 6:** GO and pathway enrichment analysis of the *M. tuberculosis* H37Rv genes that are predicted to target human TNF-alpha and IL-1~IL11 signaling pathways

TNF-Alpha	GO term ID	GO aspect	GO term name	%	Shared
MTB genes that target human gene *PSMC2*	GO:0005524	F	ATP binding	26.92	Yes
	GO:0016020	C	membrane	20.51	Yes
	GO:0055114	P	oxidation-reduction process	16.67	No
	GO:0016310	P	phosphorylation	5.98	No
	GO:0006412	P	translation	5.13	No
	GO:0055085	P	transmembrane transport	2.14	Yes
	GO:0046933	F	hydrogen ion transporting ATP synthase activity, rotational mechanism	1.71	No
Human gene *PSMC2*	GO:0002479	P	antigen processing and presentation of exogenous peptide antigen via MHC class I, TAP-dependent	–	–
	GO:0043687	P	post-translational protein modification	–	–
	GO:0045899	P	positive regulation of RNA polymerase II transcriptional preinitiation complex assembly	–	–
Targeted human genes	*CASP10,BID,RUVBL2,DDX21,RPL4,BRINP1,PSMC2,PSMD1,FANCD2,MTIF2,PSMD2,PSMB5,RPS11,PSMC3,GLUL,PDCD2,KTN1*				
IL-1 ~ IL-11	GO term ID	GO aspect	GO term name	%	Shared
M.TB genes that target the human gene *VCP*	GO:0003824	F	catalytic activity	32.17	No
	GO:0016021	C	integral to membrane	16.78	No
	GO:0005524	F	ATP binding	27.27	Yes
	GO:0055114	P	oxidation-reduction process	9.79	No
	GO:0006810	P	transport	3.50	Yes
	GO:0006281	P	DNA repair	2.10	No
Human gene *VCP*	GO:0005515	F	protein binding	–	–
	GO:0006914	P	autophagy	–	–
	GO:0016236	P	macroautophagy	–	–
	GO:0010918	P	positive regulation of mitochondrial membrane potential	–	–
	GO:0006974	P	cellular response to DNA damage stimulus	–	–

Targeted human genes BCL2L11,UNC119,IRS1,IL2,DOK2,PTPN6,BAD,IL11,VCP,IRS2

C denotes cellular component, F denotes molecular function, and P denotes biological process

#### TNF-alpha signaling pathway

The tumor necrosis factor alpha (TNF-alpha) is a pro-inflammatory cytokine that belongs to the TNF superfamily [48]. *M. tuberculosis* H37Rv is predicted to invade human TNF-alpha signaling pathway through 443 MTB-human PPIs and the targeted human genes {*CASP10, BID, RUVBL2, DDX21, RPL4, BRINP1, PSMC2, PSMD1, FANCD2, MTIF2, PSMD2, PSMB5, RPS11, PSMC3, GLUL, PDCD2, KTN1*} (see Additional file 11). Taking the targeted human gene *PSMC2* for example (see Table 6), among the *M. tuberculosis* H37Rv genes that target the human gene *PSMC2*, 20.51% of proteins are located at membrane (GO:0016020); 16.67% of genes are involved in the biological process of oxidation-reduction process (GO:0055114); 2.14% of genes are involved in the biological process of transmembrane transport (GO:0055085). Besides the GO terms marked with “shared” in Table 6, the human gene *PSMC2* also gets involved in translation (GO:0046933;hydrogen ion transporting ATP synthase activity, rotational mechanism) and post-translational protein modification (GO:0043687). Especially, the targeted human gene *PSMC2* plays an important role in the host immune response (GO:0002479; antigen processing and presentation of exogenous peptide antigen via MHC class I, TAP-dependent).

#### IL1~IL11 signaling pathway

Interleukins are a group of cytokines (secreted proteins and signal molecules) that were first seen to be expressed by white blood cells (leukocytes). The function of immune system depends in a large part on interleukins that promote the development and differentiation of T and B lymphocytes, and hematopoietic cells [53]. *M. tuberculosis* H37Rv is predicted to interact with human IL-1~ 11 signaling pathways through 157 MTB-human PPIs and the targeted human genes {*BCL2L11, UNC119, IRS1, IL2, DOK2, PTPN6, BAD, IL11, VCP, IRS2*} (see Additional file 11). Taking the targeted human gene *VCP* for example (see Table 6), among the *M. tuberculosis* H37Rv genes that target human gene *VCP*, 16.78% of genes are located at integral to membrane (GO:0016021); 27.27% of genes fulfil the molecular function of ATP binding (GO:0005524); 9.79% of genes are involved in oxidation-reduction process (GO:0055114); 3.50% of genes are involved in transport (GO:0006810); 9.79% of genes are involved in DNA repair (GO:0006281), etc. Besides ATP binding (GO:0005524) and transport (GO:0006810), the targeted human gene *VCP* gets involved in autophagy (GO:0006914), macroautophagy (GO:0016236) and cellular response to DNA damage stimulus (GO:0006974), etc.

## Conclusions

In this work, we provide a general computational framework to exploit the knowledge of the pathogen protein interaction networks in the database STRING for the rapid reconstruction of pathogen-host protein interaction networks. We take full advantage of the co-evolution relationship between *M. tuberculosis* H37Rv and *H. sapiens* to derive significant interlogs, which are used as the training data to build a predictive model. The knowledge transfer model effectively solves the problem that no experimental bacteria-host protein interactions are available as training data. The predicted protein interactions provided in the Additional files promise to gain applications in the two fields (1) providing an alternative solution to drug resistance; (2) revealing the patterns that *M. tuberculosis* H37Rv genes target human immune signaling pathways.

## Additional files

Additional file 1: Text file contains the positive training data consisting of the derived significant interlogs. (TXT 1201 kb)

Additional file 2: Text file contains the negative training data consisting of randomly sampled non-interlogs. (TXT 769 kb)

Additional file 3: Text file contains the gene ontology analysis of the derived interlogs that get involved in drug resistance of cytochromes and other target-modifying enzymes. (TXT 1703 kb)

Additional file 4: Text file contains the gene ontology analysis of the derived interlogs that get involved in drug resistance of SOS-response and DNA replication. (TXT 1157 kb)

Additional file 5: Text file contains the predicted results on the positive independent test set consisting of less significant interlogs. (TXT 1055 kb)

Additional file 6: Text file contains the predicted results on the negative independent test set consisting of randomly sampled non-interlogs. (TXT 80 kb)

Additional file 7: Text file contains the gene ontology analysis of the validated less significant interlogs that get involved in drug resistance of antibiotic efflux pumps. (TXT 234 kb)

Additional file 8: Text file contains the gene ontology analysis of the validated less significant interlogs that get involved in drug resistance of target-modifying enzymes. (TXT 21 kb)

Additional file 9: Text file contains the predicted results on the prediction set consisting of randomly sampled non-interlogs. (TXT 552 kb)

Additional file 10: Text file contains the summary of the derived or predicted M.TB-human PPIs. (TXT 2888 kb)

Additional file 11: Text file contains the human cancer/immune signaling pathways that *M. tuberculosis* H37Rv genes are predicted to target. (TXT 106 kb)

Additional file 12: Text file contains the *M. tuberculosis* H37Rv genes that target human cancer/immune signaling pathways. (TXT 21 kb)

## Abbreviations

AR: Androgen receptor
*B. anthracis*: *Bacillus anthracis*
BCR: B cell receptor
*Ecoli*: *Escherichia coli*
*F. tularensis*: *Francisella tularensis*
GO: Gene ontology
*H. sapiens*: *Homo sapiens*
IL: Interleukins are a group of cytokines
M. tuberculosis H37Rv: Mycobacterium tuberculosis
MTB: *M. tuberculosis H37Rv*
MTBC: *Mycobacterium tuberculosis complex*
RBH: Reciprocal best hits
TNF-alpha: The tumor necrosis factor alpha
TSH: Thyroid stimulating hormone
*Y. pestis*: *Yersinia pestis*
